# Malignant Transformation of a Choroidal Nevus

**DOI:** 10.7759/cureus.75056

**Published:** 2024-12-03

**Authors:** Luxi Li, Qiongyue Zhang, Peng Zhang

**Affiliations:** 1 Department of Ophthalmology, Xi'an No. 3 Hospital, the Affiliated Hospital of Northwest University, Xi'an, CHN

**Keywords:** choroid, imaging, melanoma, metastasis, nevus, treatment

## Abstract

Choroidal nevus is the most common intraocular tumor, and most cases are benign and have no symptoms. However, choroidal nevus carries a low risk for transformation into melanoma, which is a highly aggressive and deadly cancer. In this case report, we present a male patient with blurred vision in his left eye for six months. Multimodal fundus imaging reported a large pigmented gray-brown choroidal lesion adjacent to the optic disc in his left eye, along with intraretinal and subretinal fluid (SRF) accumulation involving the macula and fluorescein leakage. Choroidal nevus was then diagnosed, and bevacizumab was given by intravitreal injection. After one month of bevacizumab treatment, spectral-domain optical coherence tomography showed a decrease in SRF. After four months of bevacizumab treatment, the patient suffered from more SRF and an elevated lesion in his left eye. During subsequent follow-up, the lesion continued to increase in size. Given the possibility of a malignant transformation of choroidal nevus into melanoma, transpupillary thermotherapy (TTT) was performed. The choroidal melanoma appeared to be necrotic at 10 months post-TTT. The patient's left eye was enucleated due to neovascular glaucoma 17 months post-TTT. Histopathologic examination showed diffuse infiltration of melanoma cells in the retina, choroid and optic nerve, and cell growth on the surface of the optic nerve papilla. Multiple treatment modalities, including external beam radiotherapy and systemic chemotherapy, were applied, but the patient eventually died due to metastasis. Patients with choroidal nevus should be managed with multimodal fundus imaging and be closely followed up due to the risk of transformation into a malignant melanoma. For cases with clinical and imaging features of malignant transformation, early intervention and long-term follow-up are required to achieve local disease control and vision preservation.

## Introduction

Choroidal nevus is the most common intraocular tumor, and the prevalence of choroidal nevi in a healthy population using a scanning laser ophthalmoscope is 10%; most cases are benign and have no symptoms, and most cases are discovered during physical examination [1]. However, choroidal nevus carries a low risk for transformation into malignant melanoma, and the annual rate of malignant transformation of a choroidal nevus was estimated to be 1 in 8845 [2]. Clinical presentation of choroidal melanoma may be non-specific and includes metamorphopsia, progressive visual field loss, and blurry vision. Choroidal melanoma is often diagnosed clinically during fundus examination. Patients with choroidal melanoma are at lifetime risk of having metastasis even with treatment of primary malignancy [1,2]. Suppose a choroidal nevus lesion has a large basal area and has clinical signs such as subretinal fluid (SRF), tumor thickness >2 mm, and visual acuity loss of 20/50 or worse. In that case, it should be highly suspected that the choroidal nevus is undergoing a malignant transformation [1-3]. Other conditions that increase the incidence of choroidal melanomas are ultraviolet exposure, mutational inactivation of the (BRCA1-associated protein 1) BAP 1 tumor-suppressor gene, and oculodermal melanocytosis [4].

Here, we present a case of choroidal nevus with a large basal area that transformed into a malignant melanoma. Multiple treatment modalities, including eyeball enucleation, external beam radiotherapy, and systemic chemotherapy, were administered, but the patient ultimately died due to metastasis. This case report aims to illustrate the clinical course of a choroidal nevus transforming into melanoma and underscore lessons in managing and monitoring such cases.

## Case presentation

**Figure 1:**
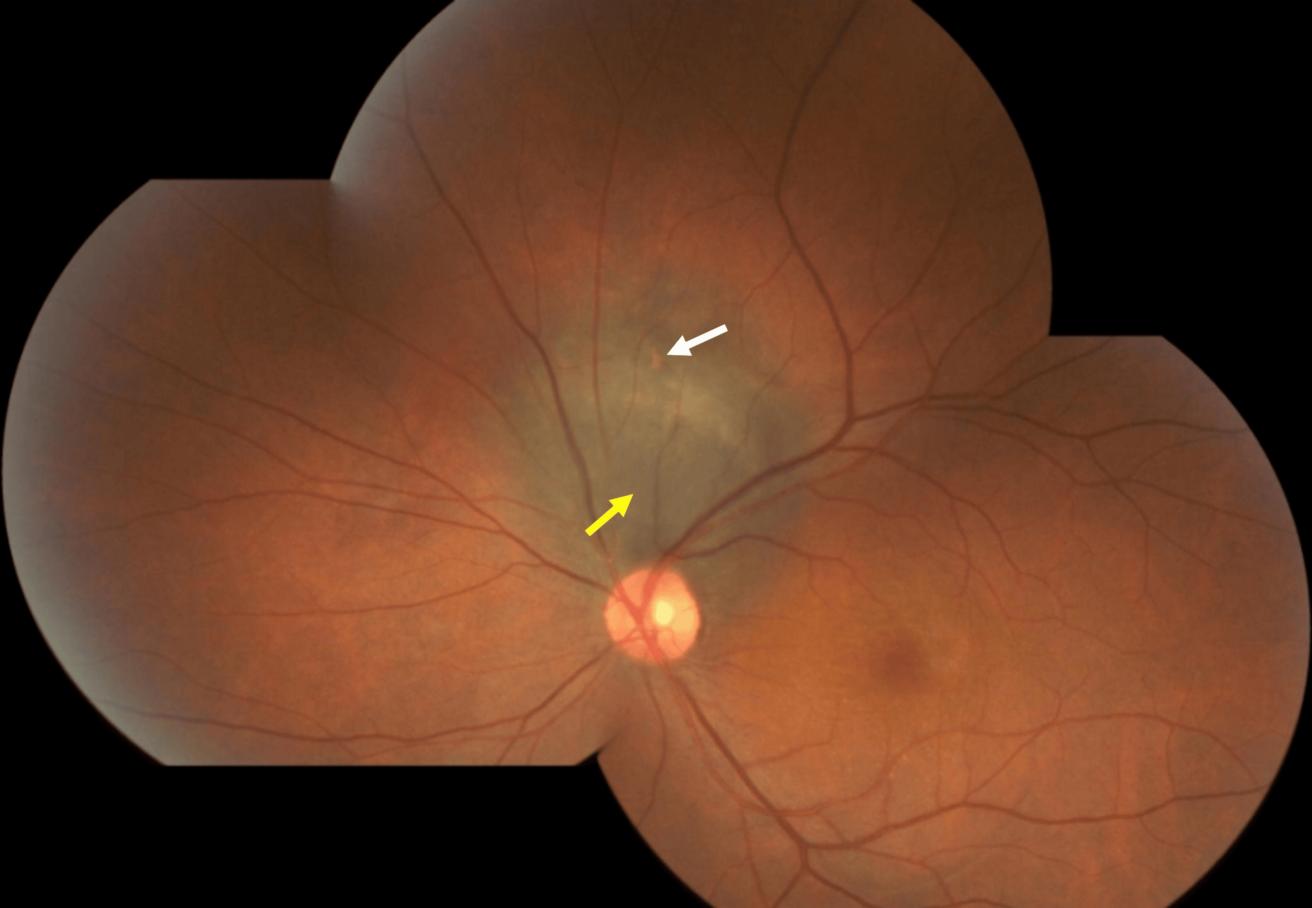
Color fundus photography shows a large pigmented gray-brown choroidal lesion (yellow arrow) adjacent to the optic disc with prominent overlying orange pigment (white arrow)

**Figure 2:**
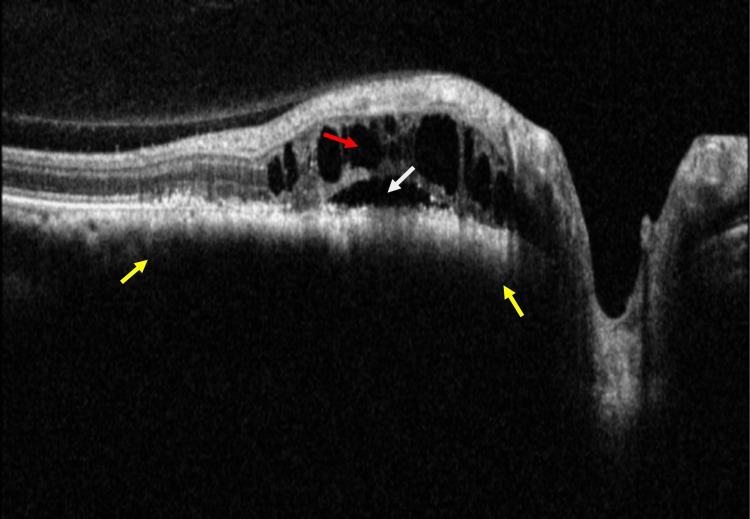
Spectral-domain optical coherence tomography (SD-OCT) shows hyperreflective front surface, choroidal shadowing due to choroidal lesion (yellow arrows), subretinal fluid (SRF) (white arrow), and intraretinal fluid (IRF) (red arrow) over the choroidal lesion

**Figure 3:**
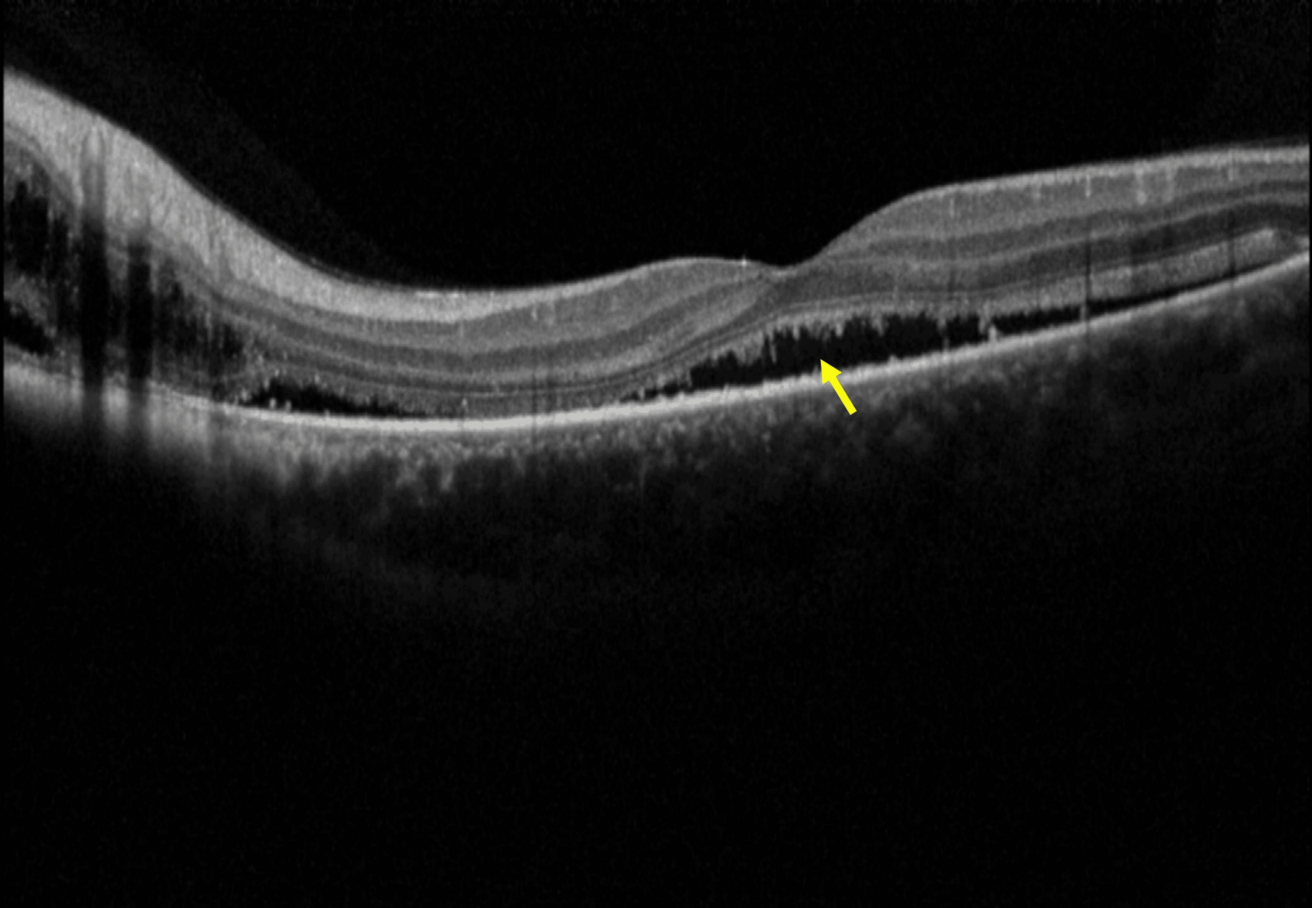
Spectral-domain optical coherence tomography (SD-OCT) shows subretinal fluid (SRF) (yellow arrow) involving the fovea

**Figure 4:**
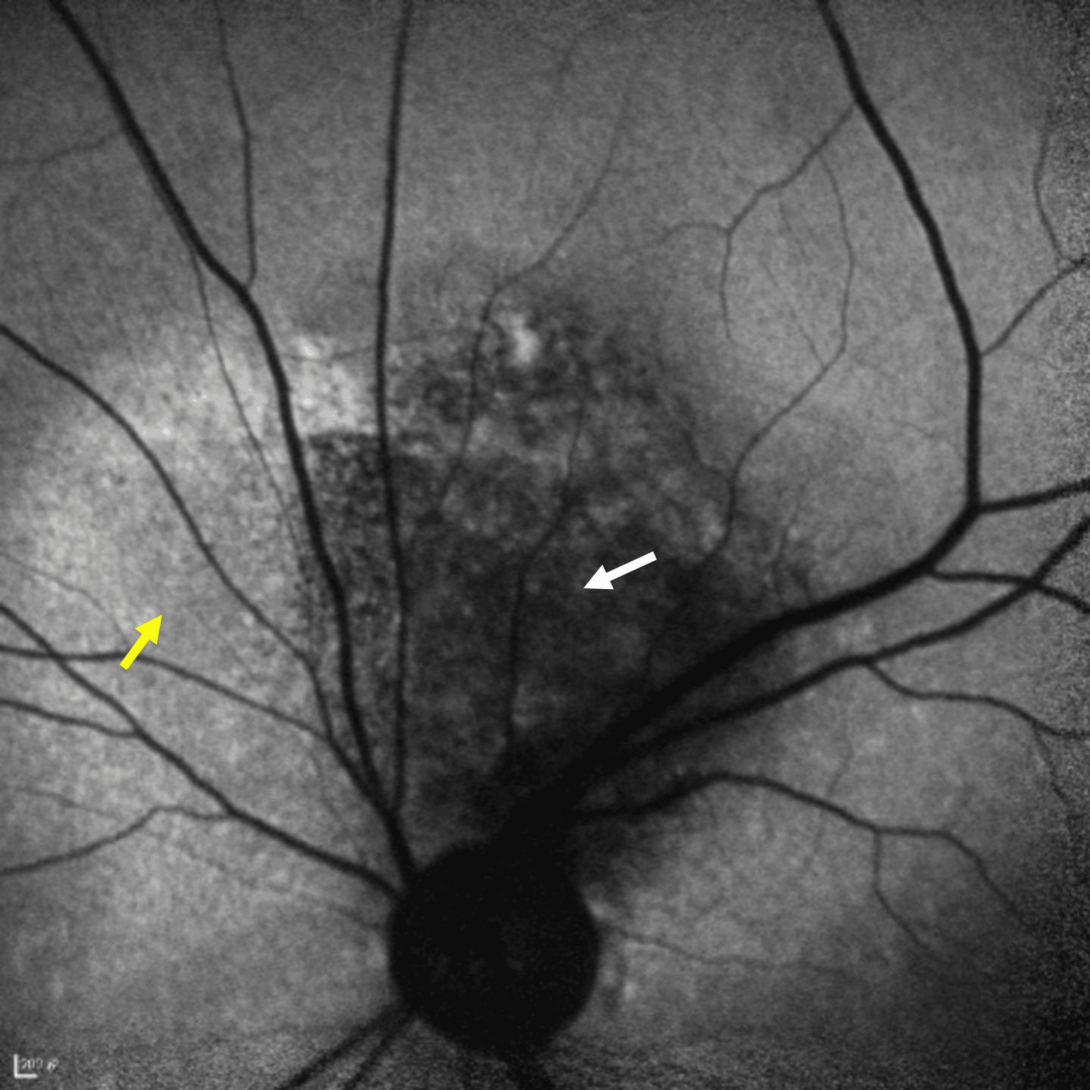
Short-wave autofluorescence (SW-AF) imaging shows a hypo-fluorescent area with an indistinct border (white arrow), surrounded by a mottled halo of hyper-fluorescent (yellow arrow)

**Figure 5:**
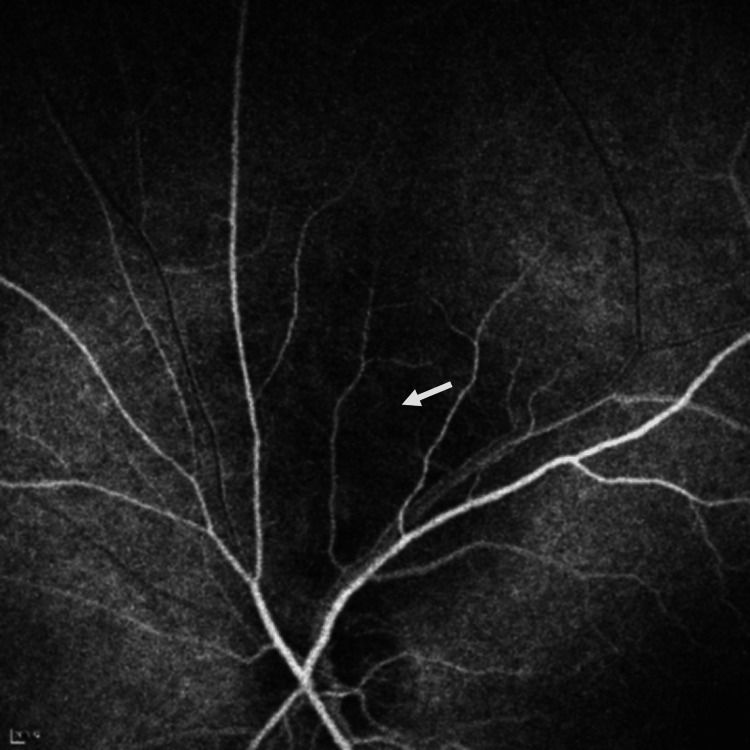
Fluorescein angiography (FA) shows local hypo-fluorescence (white arrow) in the early phase

**Figure 6:**
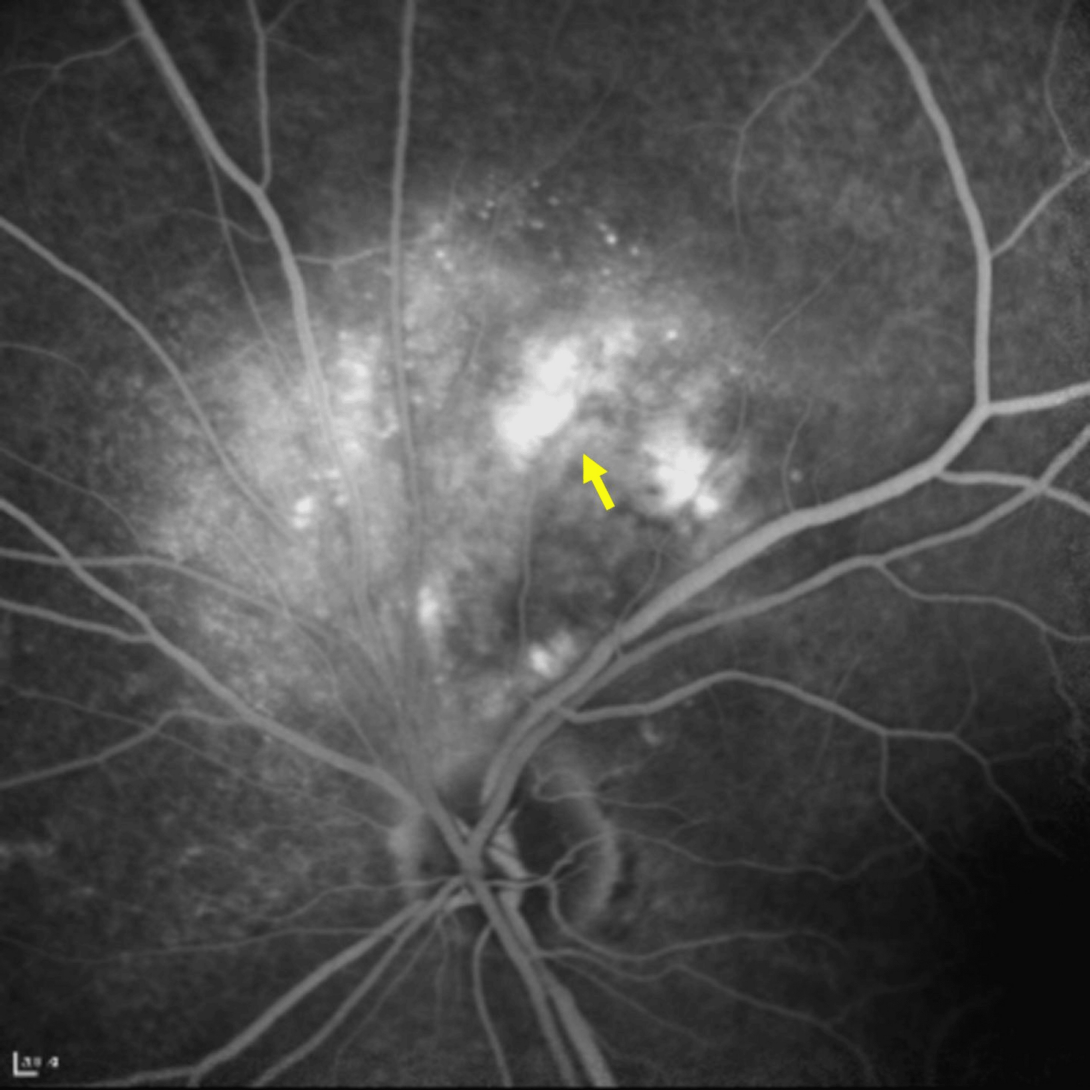
Fluorescein angiography (FA) shows diffuse fluorescein leakage over the choroidal lesion (yellow arrow) in late phase

**Figure 7:**
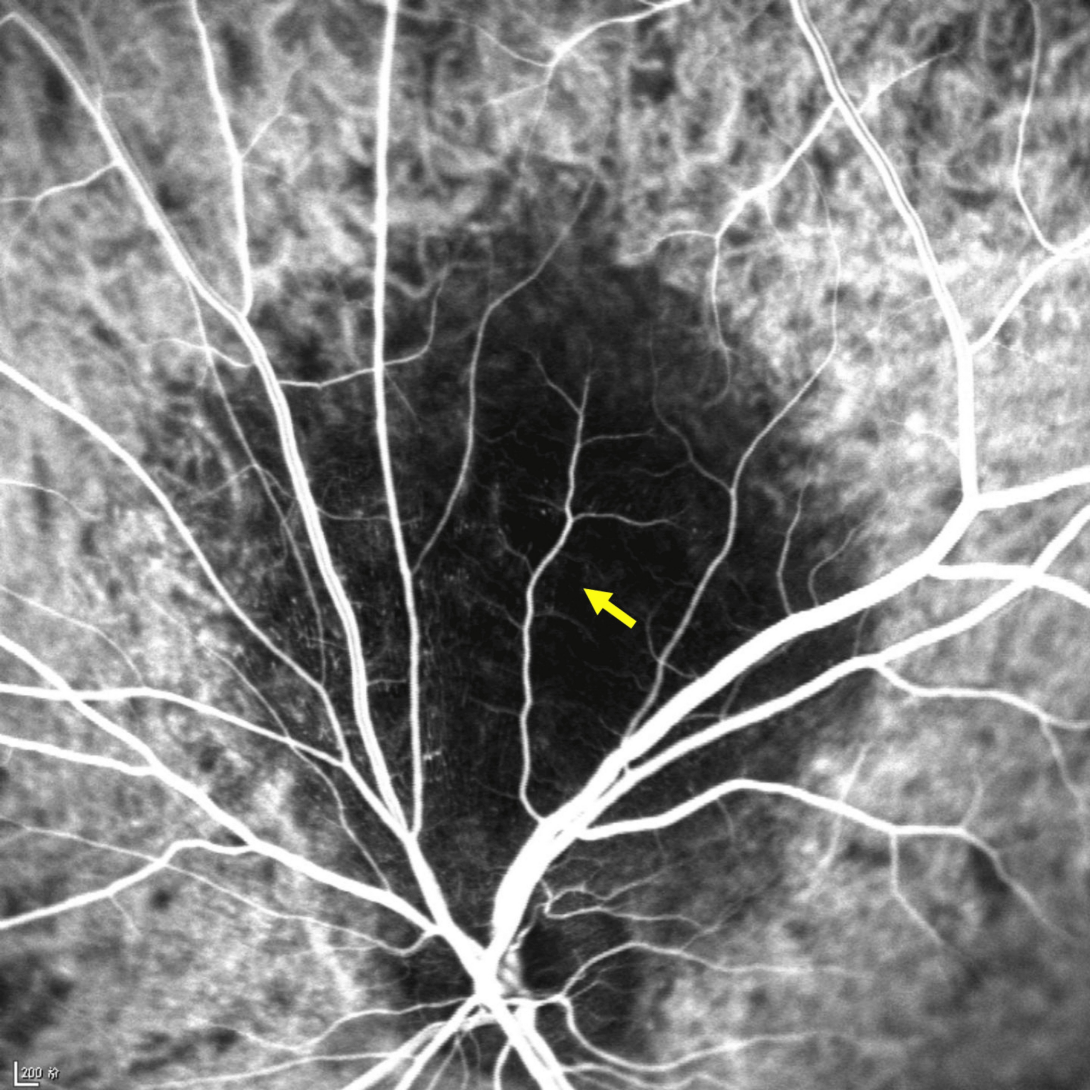
Indocyanine green angiography (ICGA) illustrates local hypo-fluorescence (yellow arrow) under the retina

In March 2016, a 45-year-old male patient presented to our ophthalmology clinic with blurred vision in his left eye for six months. His best corrected visual acuity (BCVA) was 20/40 and 20/20 in the left eye and right eye, respectively. The anterior segment and intraocular pressure of both eyes were normal. Fundus examination reported a large pigmented gray-brown choroidal lesion (with prominent overlying orange pigment) adjacent to the optic disc in the left eye (Figure 1). Spectral-domain optical coherence tomography (SD-OCT) of the lesion (red star) showed a hyper-reflective front surface, choroidal shadowing, SRF, and intraretinal fluid (IRF) over the choroidal lesion (Figure 2). SRF was found to involve the fovea (Figure 3). Short-wave autofluorescence (SW-AF) imaging showed a hypo-fluorescent area with an indistinct border, surrounded by a mottled halo that was hyper-fluorescent (Figure 4). Fluorescein angiography (FA) showed local hypo-fluorescence in the early phase (Figure 5) and diffuse fluorescein leakage over the choroidal lesion in the subsequent phases (Figure 6). Indocyanine green angiography (ICGA) illustrated hypo-fluorescence under the retina (Figure 7), consistent with a choroidal lesion.

Based on multimodal imaging features, SRF associated with choroidal nevus was diagnosed. Due to IRF, SRF, and fluorescein leakage over the choroidal lesion, anti-human vascular endothelial growth factor A (VEGF-A), a monoclonal antibody bevacizumab was given by intravitreal injection in the left eye.

**Figure 8:**
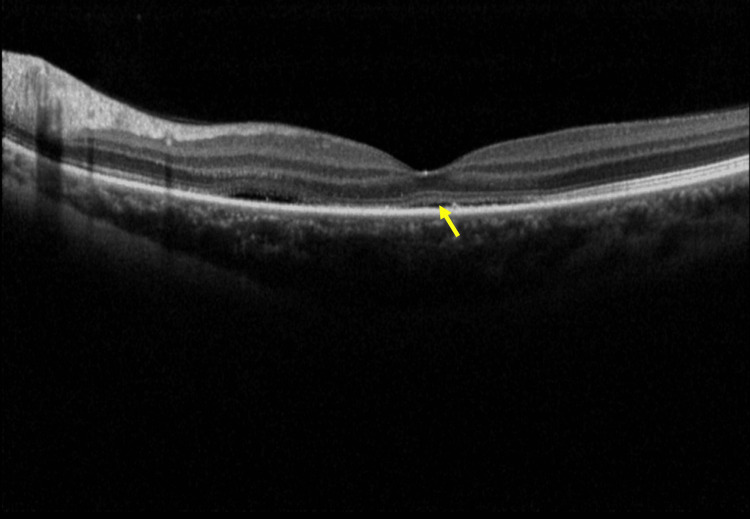
Spectral-domain optical coherence tomography (SD-OCT) shows a significant reduction of subretinal fluid (SRF) (yellow arrow) in the macula

**Figure 9:**
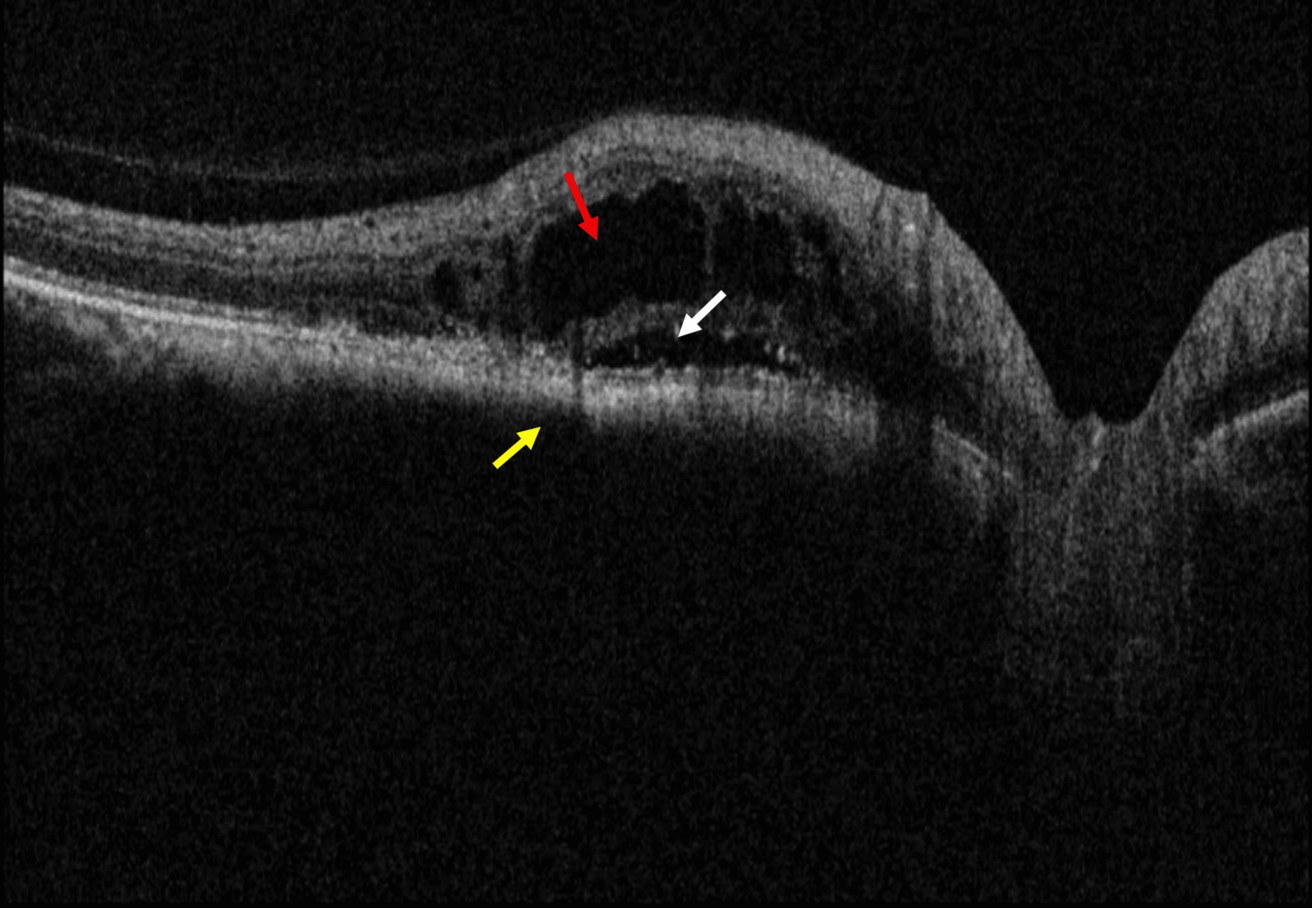
Spectral-domain optical coherence tomography (SD-OCT) shows intraretinal fluid (IRF) (red arrow) and subretinal fluid (SRF) (white arrow) persist in the corresponding area of the choroidal lesion (yellow arrow)

After one month of bevacizumab treatment (April 2016), the BCVA of the left eye improved to 6/10. SD-OCT showed a significant decrease in SRF in the macula (Figure 8). However, IRF and SRF persisted in corresponding areas of the choroidal lesion (Figure 9).

**Figure 10:**
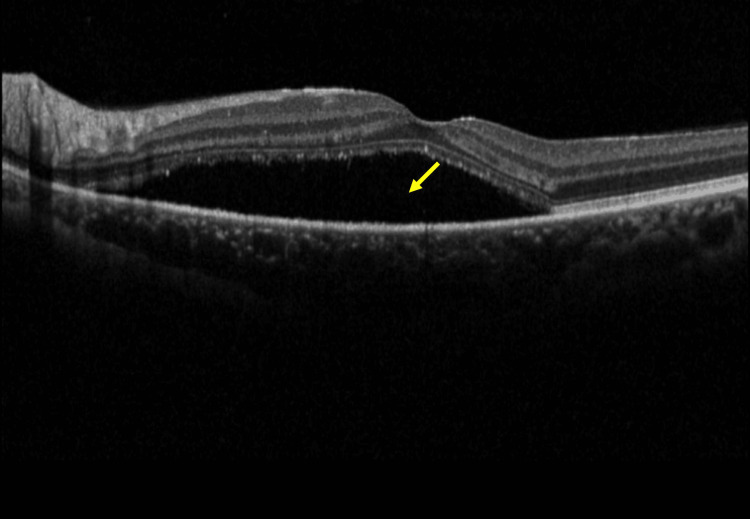
Spectral-domain optical coherence tomography (SD-OCT) shows an increased subretinal fluid (SRF) (yellow arrow) in the macula

**Figure 11:**
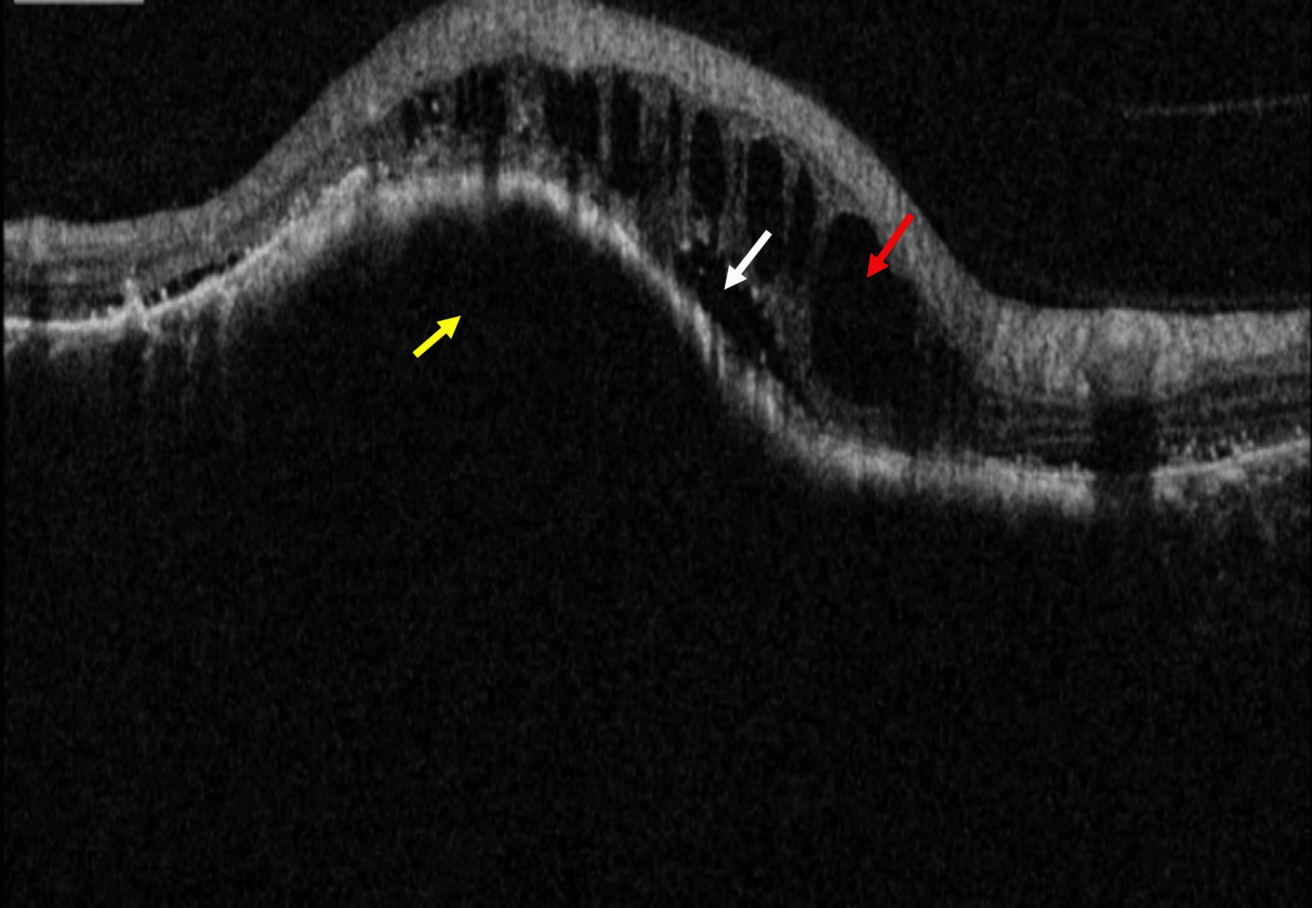
Spectral-domain optical coherence tomography (SD-OCT) shows the increased protrusion of choroidal lesion (yellow arrow), intraretinal fluid (IRF) (red arrow), and subretinal fluid (SRF) (white arrow) in the corresponding area of choroidal lesion

In July 2016, after four months of bevacizumab treatment, the patient returned to our clinic, and the BCVA of the left eye was decreased to 20/40. SD-OCT showed more SRF accumulated in the macula (Figure 10). The degree of protrusion of the choroidal lesion was increased, and IRF and SRF persisted in corresponding areas of the choroidal lesion (Figure 11). Based on clinical manifestations, malignant transformation was considered in the choroidal lesion. Due to the position and large area of the lesion, radiation therapy or enucleation was recommended. However, the patient refused and requested the follow-up to continue.

**Figure 12:**
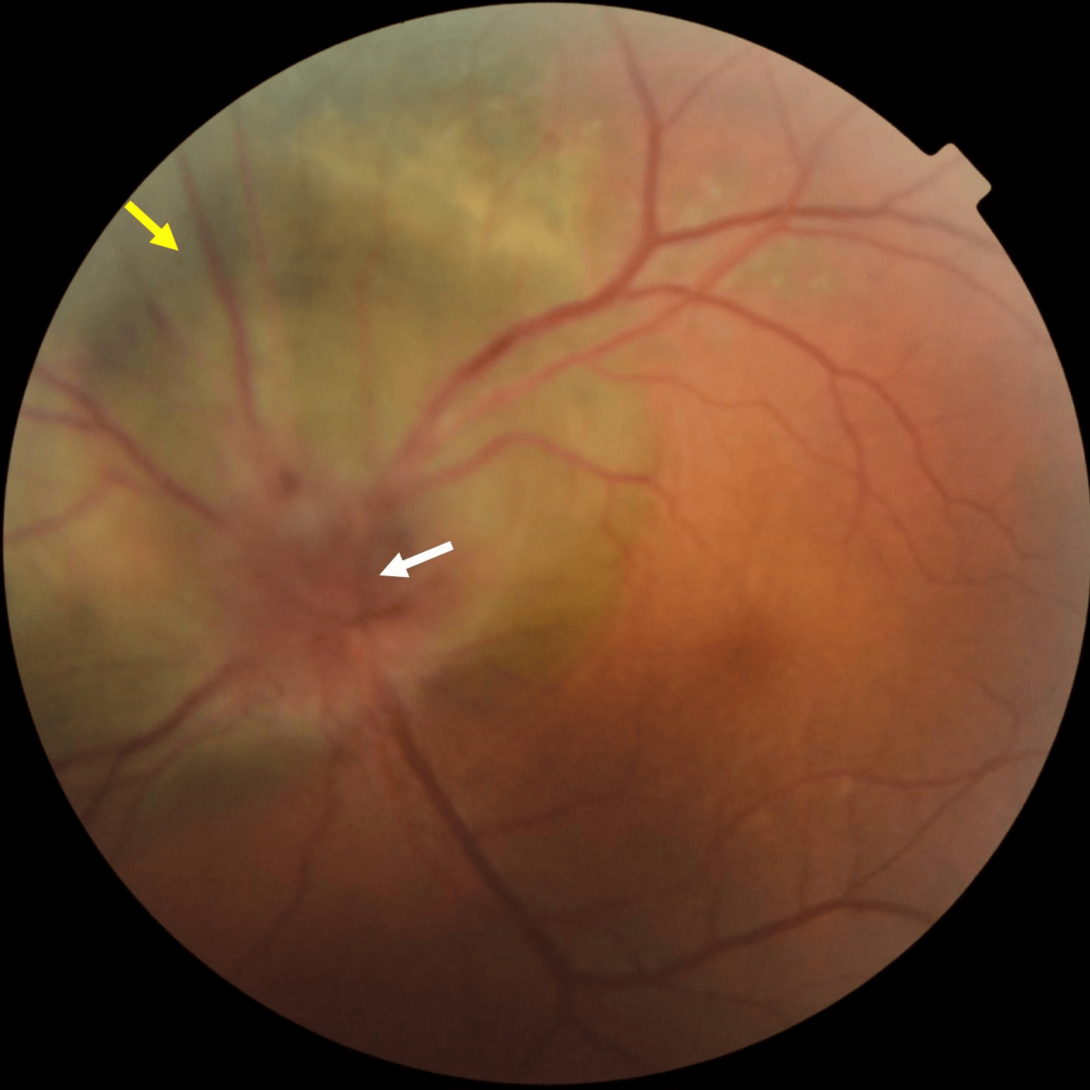
Color fundus photography shows tortuous and dilated retinal veins and optic nerve papilla edema (white arrow), the choroidal lesion (yellow arrow) enlarging and growing around the optic nerve papilla

In January 2017, the patient underwent a follow-up examination and reported a continuous decline in his left eye vision. The BCVA of the left eye was counting fingers/30 cm. Ophthalmoscopically, retinal veins in the left eye were tortuous and dilated, and optic nerve papilla edema was obvious. The choroidal lesion enlarged and grew around the optic nerve papilla (Figure 12). Treatment like enucleation was recommended to be performed as soon as possible, considering the cosmetic impact of losing an eye. The patient refused enucleation and requested further consultations in other hospitals.

**Figure 13:**
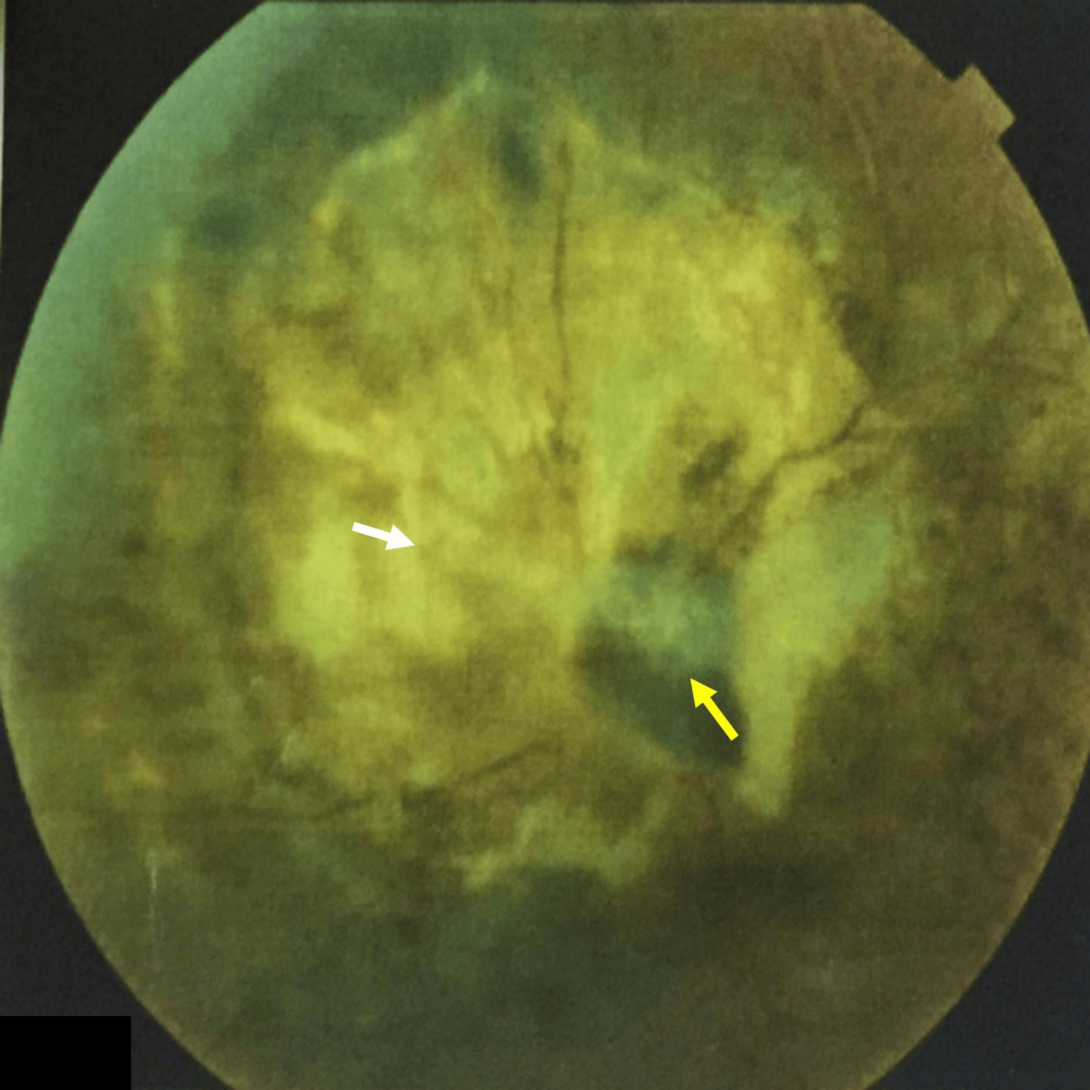
Color fundus photograph shows the retinal arteries and veins in the left eye displaying attenuation and a black lesion (yellow arrow) on the surface of the optic nerve papilla. The original choroidal lesion appeared as a flat gray-white lesion (white arrow)

In November 2017, the patient came to our ophthalmology clinic for a follow-up examination. As the patient considered the transpupillary thermotherapy (TTT) procedure to be simple and cost-effective, he self-reported receiving a TTT nine months ago at another hospital's ophthalmology department. The vision was light perception (LP) in his left eye. Ophthalmoscopically, the retinal arteries and veins in the left eye displayed attenuation, and there was a black lesion about one optic disc diameter in size on the surface of the optic nerve papilla. The original choroidal lesion appeared as a flat gray-white lesion, which was considered to be a manifestation of necrosis (Figure 13). A close follow-up was suggested.

**Figure 14:**
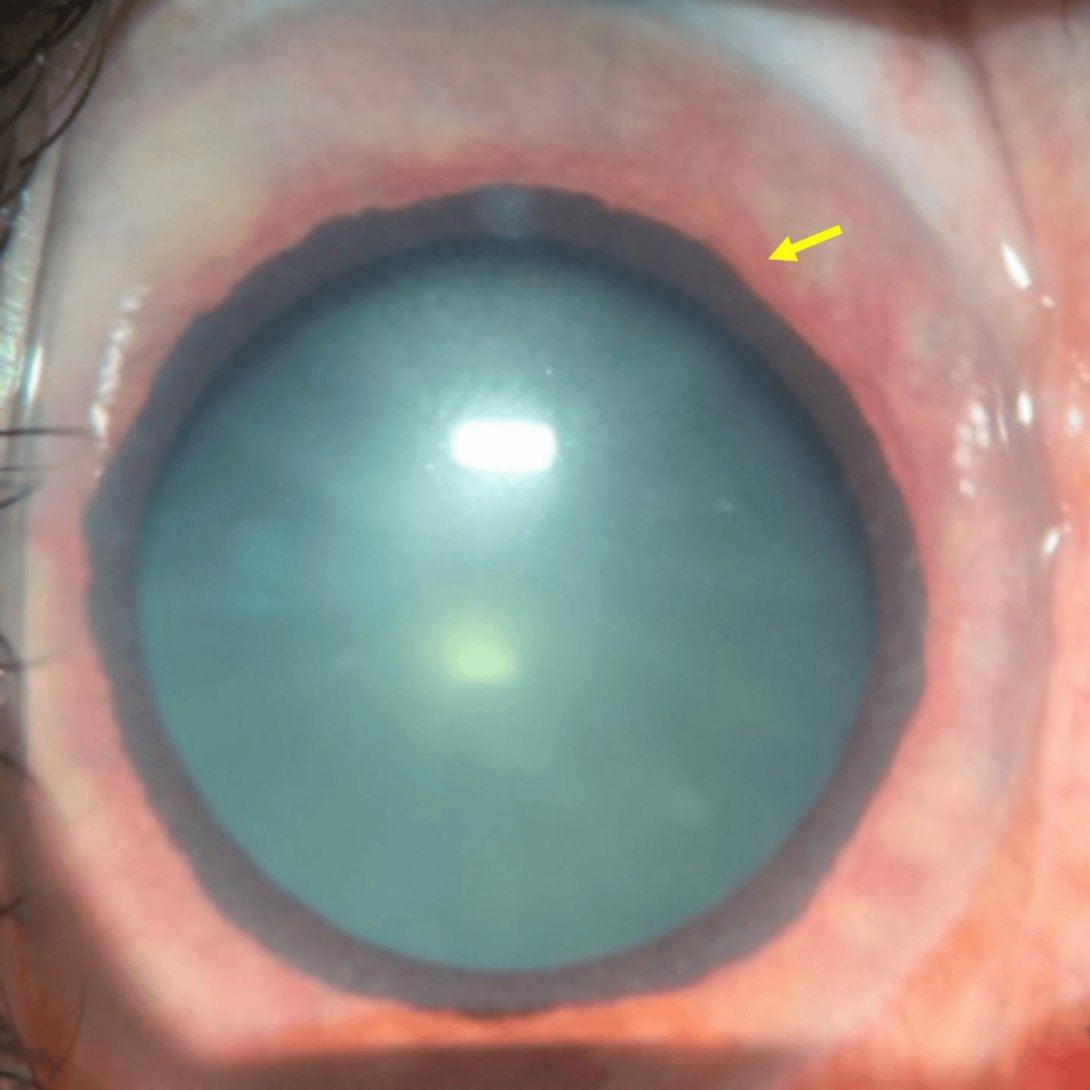
Slit-lamp anterior-segment photography shows pupil dilation and massive iris neovascularization (yellow arrow)

**Figure 15:**
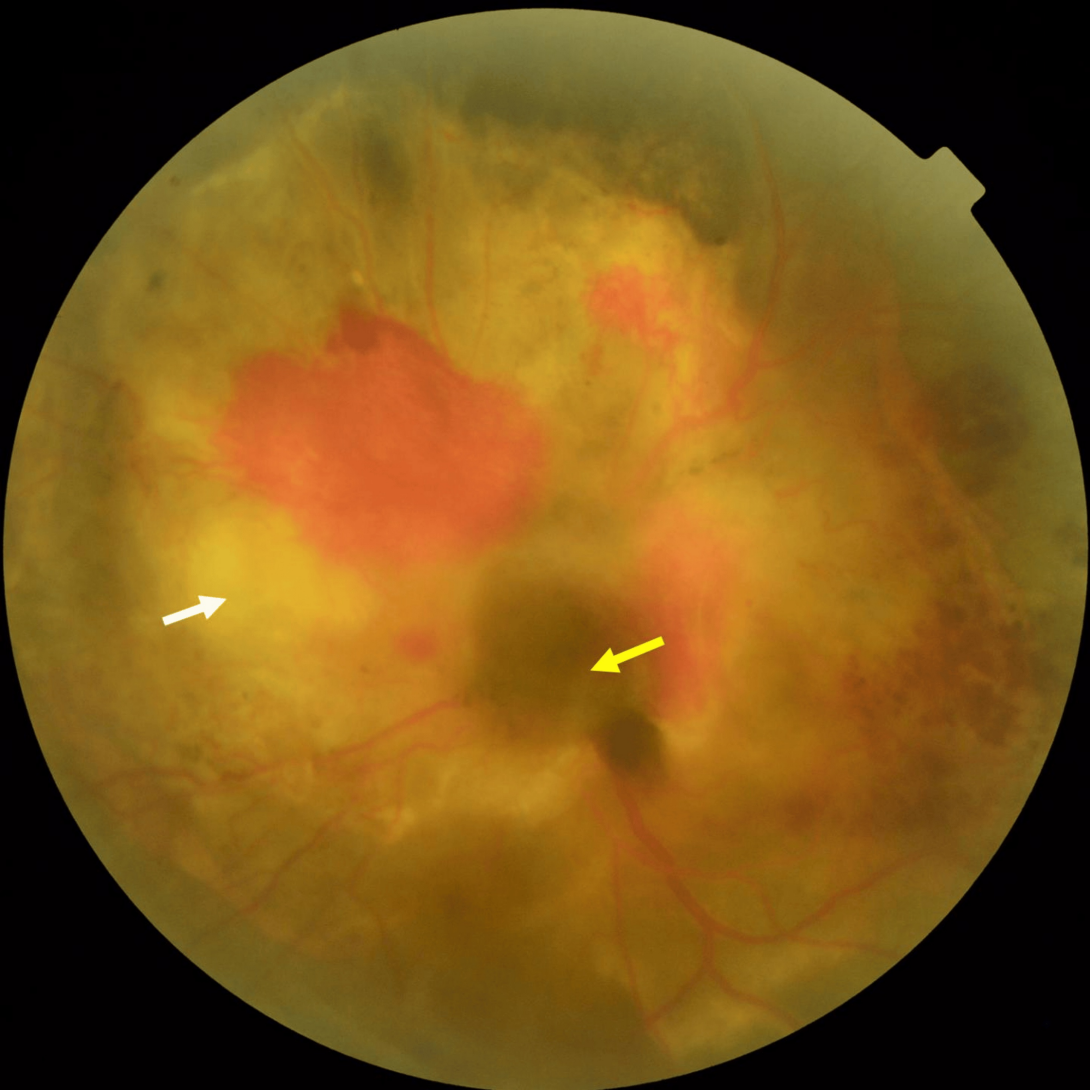
Color fundus photography shows an indistinct melanoma (yellow arrow) on the surface of the optic nerve papilla with retinal hemorrhage and dilated retinal veins and a yellow-white, poorly circumscribed lesion (white arrow) under the retina around the optic nerve papilla

**Figure 16:**
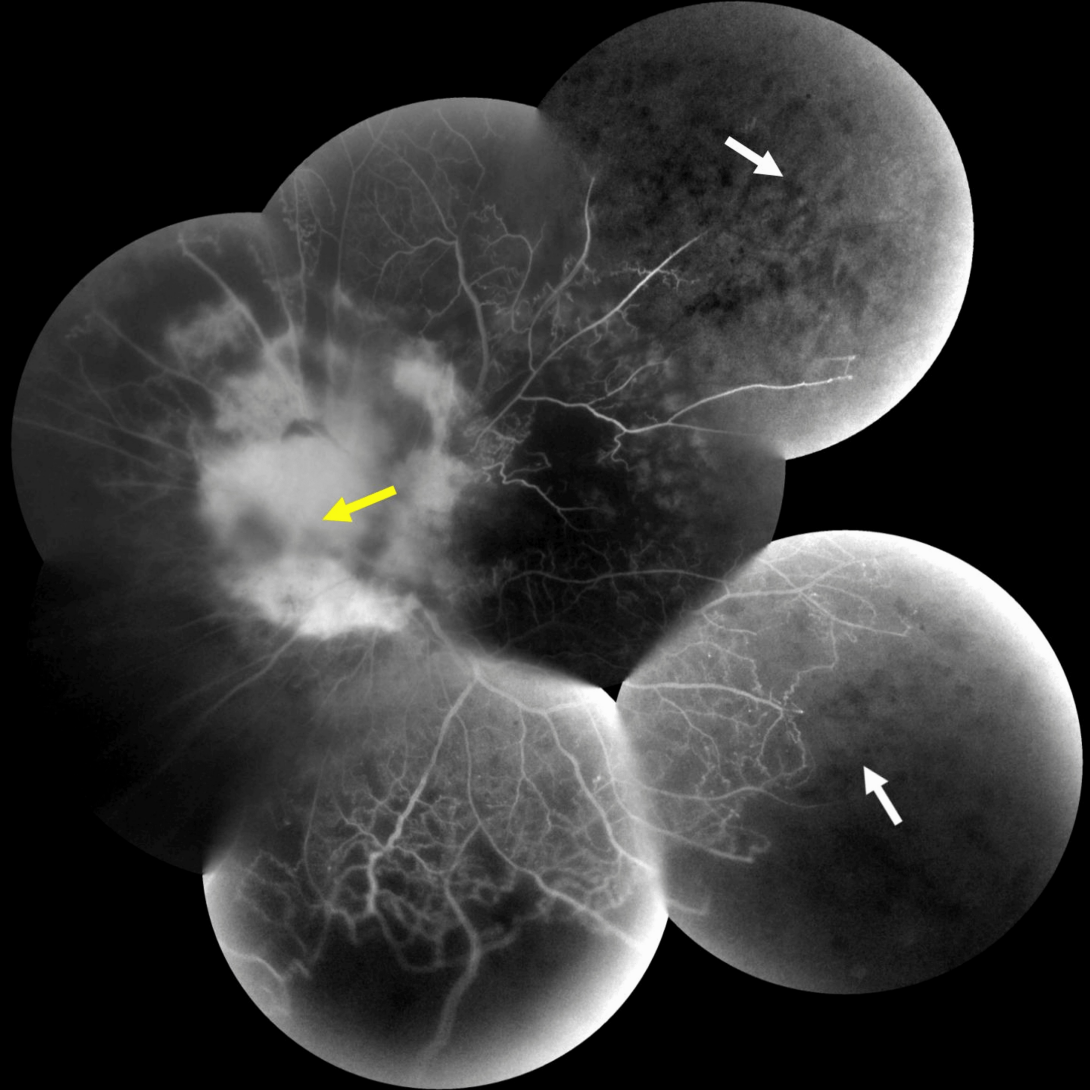
Fluorescein angiography (FA) shows hyper-fluorescence with unclear boundaries (yellow arrow) around the optic nerve papilla and extensive non-perfusion area (white arrow)

In June 2018, the patient returned to our clinic due to left eye swelling and pain with no LP. Intraocular pressure of the left eye was 55 mmHg. The pupil of the left eye was dilated, and massive iris neovascularization was found (Figure 14). The lesion on the surface of the optic disc grew indistinctly, accompanied by retinal hemorrhage and dilated retinal veins. In addition, a yellow-white, poorly circumscribed lesion under the retina around the optic nerve papilla was found in the left eye (Figure 15). FA showed hyper-fluorescence with unclear boundaries around the optic nerve papilla and extensive retinal non-perfusion (Figure 16). The patient was diagnosed with neovascular glaucoma, central retinal vein occlusion, and choroidal melanoma in his left eye.

**Figure 17:**
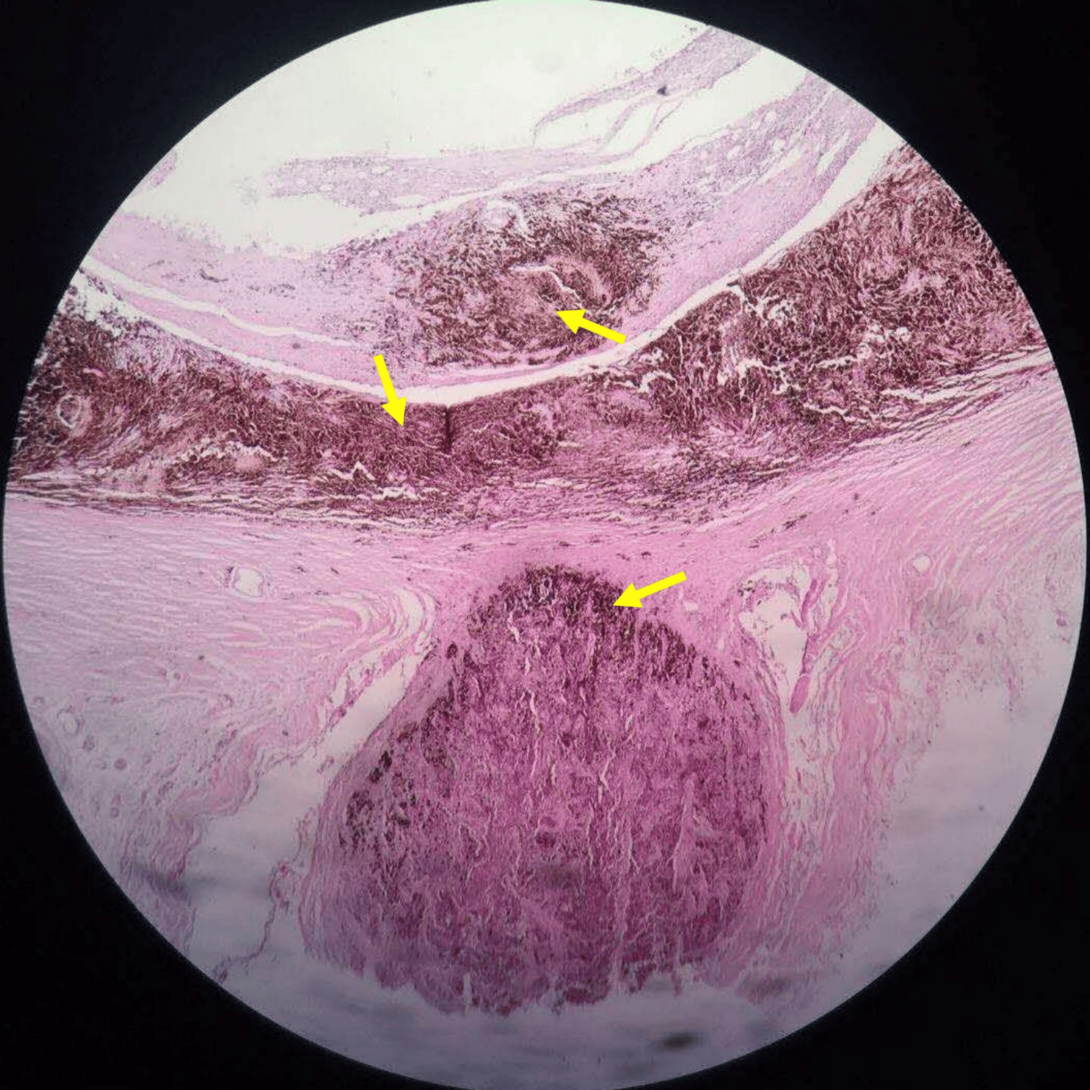
Hematoxylin and eosin (H&E) staining shows diffuse infiltration of melanoma cells (yellow arrow) in the retina, choroid and optic nerve, and melanoma cell growth on the surface of the optic papilla

Enucleation of the patient’s left eye and hydroxyapatite artificial eye implantation were performed after obtaining informed consent. The enucleated eyeball was subjected to pathological examination. Hematoxylin and eosin (H&E) staining of tissue sections located at the posterior pole of the eyeball showed diffuse infiltration of melanoma cells in the retina, choroid and optic nerve, and cell growth on the surface of the optic papilla (Figure 17). The melanoma cells were spindle-shaped and epithelioid-mixed cells with large and protruding nucleoli and abundant melanin in the cytoplasm.

As the optic nerve was already infiltrated with melanocytes, palliative treatment including adjuvant external beam radiotherapy and chemotherapy (carboplatin, dacarbazine, and interferon) at an ocular oncology specialist was recommended.

In February 2019, the patient died due to intracranial and systemic melanoma metastases even after receiving external beam radiotherapy and chemotherapy.

## Discussion

Choroidal nevus is a common intraocular tumor, with a prevalence of 5.6% in the White population, 2.7% in the Hispanic population, and 0.6% in the Black population [3]. Although choroidal nevus usually remains stable over time, it carries a low risk for transformation into malignant melanoma, estimated at one in 8845 annually [2]. Patients with choroidal nevus typically have no ocular symptoms. However, melanomas often exhibit rapid growth, which may cause visual disturbances and thus prompt patients to seek medical attention [2,3].

The mnemonic "To Find Small Ocular Melanoma Doing Imaging" (TFSOM-DIM), based on clinical and imaging features (risk factors) that predict the risk of a nevus transforming into a melanoma, stands for thickness >2 mm (by ultrasonography), subretinal effusion (by OCT), symptoms of vision loss, orange pigment (by autofluorescence), melanoma cavity (by ultrasonography), and diameter >5 mm [5,6]. The five-year estimates for nevus growth into melanoma were found to be 1% with no risk factors, 11% with one factor, 22% with two factors, and 34% with three or more factors [7,8]. Although this mnemonic is a useful clinical tool, we must be aware that it has limitations in terms of prediction and is part of an evolving set of risk factors rather than an absolute diagnostic tool.

On the first visit to our ophthalmology clinic, the patient's choroidal lesion was already large and accompanied by IRF and SRF. Additionally, the lesion was found adjacent to the optic nerve. In this case, the risk of malignant transformation into melanoma was already high according to TFSOM-DIM.

Choroidal melanoma represents ∼85% of all ocular melanomas, and up to 50% of patients develop metastatic disease [9]. Common metastatic sites include liver (89%), lungs (29%), bone (17%), skin and subcutaneous tissues (12%), and lymph nodes (11%) [10]. Choroidal melanoma is fatal in about 50% of patients [10].

In a study of choroidal melanoma patients with a median observation period of 28 years, the Kaplan-Meier estimator of metastasis occurrence was 32% by five years, 50% by 15 years, 56% by 25 years, and 62% by 35 years. In addition, of the patients who died of choroidal melanoma, 90% died within 15 years and 98% died within 25 years [11].

The patient reported here suffered from malignant transformation of choroidal nevus and initially presented to our ophthalmology clinic for vision loss due to SRF involvement in the macula. Intraocular injection of an anti-VEGF drug, bevacizumab, only improved the SRF for a short period of time but had no therapeutic value for the tumor itself. In this case, the choroidal melanoma grew rapidly during follow-up and compressed the central retinal vein, resulting in optic nerve papilla edema and tortuous and dilated veins. The seemingly necrotic tumor continued to infiltrate and grow even after treatment with TTT, leading to central retinal vein occlusion and neovascular glaucoma. Adjuvant external beam radiotherapy and systemic chemotherapy were performed after enucleation, but the patient eventually died due to melanoma metastasis.

For this patient in this case, the multimodal images showed persistent IRF and SRF, a large and persistently elevated nevus, and proximity to the optic disc; these features are all consistent with TFSOM-DIM.

Eventually, histopathology confirmed the diagnosis of choroidal melanoma and showed that the tumor was composed of spindle-shaped and epithelioid mixed cells. This means that the tumor is a mixed-cell melanoma. The morphology of the melanoma cells also has important prognostic implications for choroidal melanoma [12]. Various studies have established that a large mean diameter of nucleoli is associated with poor prognosis, and the prognosis worsens with an increasing number of epithelioid cells per high-power field. The 15-year mortality of patients with melanomas of mixed cell type is three times that of patients with tumors of pure spindle cell type [13].

Therefore, in addition to a close follow-up, TFSOM-DIM should also be referenced in conjunction with multimodal fundus imaging. A choroidal nevus that is highly suspected of having malignant changes should be intervened early.

TTT is a noninvasive treatment where infrared diode 810 nm laser light is delivered through a dilated pupil to the choroidal tumor surface [14]. This raises tumor temperature to 45-60°C, which may cause thermal cytotoxic damage and then cell apoptosis. In addition, TTT can lead to heat-induced closure of vascular networks in tumors. Eventually, the tumor managed by TTT becomes necrotic [15].

However, the 810-nm laser light has a maximum penetration depth of 4 mm and is therefore only suitable for the treatment of small pigmented choroidal melanomas (≤2.5 mm) [14].

A previous study reported that the Kaplan-Meier estimator of choroidal melanoma recurrence was 11% at five years and 15% at 10 years after primary TTT [16]. This may be related to the large size and high thickness of the tumor [15]. In addition, the Kaplan-Meier estimator of melanoma recurrence at 10 years was 18% with one or two risk factors, 35% with three to five risk factors, and 55% with >6 risk factors. Thus, TTT is less preferable in cases with multiple risk factors [14]. Treatment options for smaller choroidal melanomas include photodynamic therapy, plaque radiotherapy, proton beam radiotherapy, and local resection [4,15]. For choroidal melanomas with a basal diameter of more than 18 mm and a thickness greater than 12 mm, or with moderate extraocular extension, enucleation is recommended [16].

In recent years, targeted therapy with BRAF/MEK inhibitors and immunotherapy with PD-1, PD-L1, and CTLA-4 inhibitors have made significant strides in the outcomes and prognosis of melanoma [17].

Novel immune-based approaches, such as immune checkpoint blockade, tumor-infiltrating lymphocytes, and genetically engineered T-lymphocytes like chimeric antigen receptor (CAR) T cells, have been used in the treatment of melanoma [17,18]. Among these approaches, CAR T cell therapy has shown clinical promise and theoretical advantages in mobilizing inflammatory cells and overcoming the immune-suppressive tumor microenvironment of solid tumors and metastases [19,20].

Although immunotherapy has dramatically changed the treatment approach to cutaneous melanoma, its success in choroidal melanoma has been much more limited, which may be attributed to the intraocular immune-privileged microenvironment with inhibited antitumor immunity [20]. The intraocular tissue specificities involved in immunomodulation are mostly unknown, and more studies are needed to improve the effects of immunotherapy on choroidal melanoma.

## Conclusions

A choroidal nevus is a common ocular tumor, and patients with choroidal nevus should be closely followed up and subjected to multimodal fundus imaging due to its potential for malignant transformation. For choroidal nevus showing a propensity for malignant transformation (referring to TFSOM-DIM), early intervention and closer long-term follow-up should be carried out so as to achieve local disease control and vision preservation. However, multimodal imaging findings and TFSOM-DIM can only predict the risk of a choroidal nevus transforming into melanoma and cannot be used as a diagnostic tool for choroidal melanoma. Treatment of choroidal melanoma should also be individualized based on an in-depth assessment of the lesion and the patient's general condition, as well as a thorough evaluation of the limitations of different treatment options. Due to the lethal nature of choroidal melanoma, the patient must be educated about the expected vision outcome, risk of potential metastases, life expectancy, and management modalities.
